# Baicalin Mitigates Cardiac Hypertrophy and Fibrosis by Inhibiting the p85a Subunit of PI3K

**DOI:** 10.3390/biomedicines13010232

**Published:** 2025-01-19

**Authors:** Lu He, Min Zhu, Rui Yin, Liangli Dai, Juan Chen, Jie Zhou

**Affiliations:** 1 Department of Biochemistry and Molecular Biology, School of Basic Medicine and the Collaborative Innovation Center for Brain Science, Tongji Medical College, Huazhong University of Science and Technology, Wuhan 430022, China; d202282184@hust.edu.cn (L.H.); mzhu@tjh.tjmu.edu.cn (M.Z.); yinrui75426@163.com (R.Y.); dailiangli2002@163.com (L.D.); 2 Division of Neonatology, Department of Pediatrics, Tongji Hospital, Tongji Medical College, Huazhong University of Science and Technology, Wuhan 430022, China; 3 Department of Thoracic Surgery, Tongji Hospital, Tongji Medical College, Huazhong University of Science and Technology, Wuhan 430022, China

**Keywords:** heart failure, baicalin, PI3K, isoprenaline, transverse aortic constriction

## Abstract

**Background:** Heart failure (HF) is a serious public health concern. Baicalin is one of the major active ingredients of a traditional Chinese herbal medicine, Huang Qin, which is used to treat patients with chest pain or cardiac discomfort. However, the underlying mechanism(s) of the cardioprotective effect of baicalin are still not fully understood. **Methods:** Isoprenaline injection or transverse aortic constriction-induced animal models and isoprenaline or angiotensin 2 administration-induced cell models of heart failure were established. Baicalin (15 mg/kg/day or 25 mg/kg/day) was administered in vivo, and 10 μM baicalin was administered in vitro. Potential pharmacological targets of baicalin and genes related to heart failure were identified via different databases, which suggested that PI3K–Akt may be involved in the effects of baicalin. Molecular docking was carried out to reveal the effect of baicalin on p85a. **Results:** We observed significant antihypertrophic and antifibrotic effects of baicalin both in vivo and in vitro. The mean cross-sectional area of cardiomyocytes recovered from 390 μm^2^ in the HF group to 195 μm^2^ in the baicalin-treated group. The area of fibrosis was reduced from 2.8-fold in the HF group to 1.62-fold in the baicalin-treated group. Baicalin displayed a significant cardioprotective effect via the inhibition of the PI3K signaling pathway by binding with five amino acid residues of the p85a regulatory subunit of PI3K. The combination treatment of baicalin and an inhibitor of PI3K p110 demonstrated a stronger cardioprotective effect. The mean ejection fraction increased from 54% in the baicalin-treated group to 67% in the combination treatment group. **Conclusions:** Our work identified baicalin as a new active herbal ingredient that is able to treat isoprenaline-induced heart dysfunction and suggests that p85a is a pharmacological target. These findings reveal the significant potential of baicalin combined with an inhibitor of PI3K p110 for the treatment of heart failure and support more clinical trials in the future.

## 1. Introduction

Heart failure (HF) is a growing public health concern that affects 3–5% of the world’s population [1]. Various cardiac diseases, including hypertension, coronary heart disease, myocarditis, etc., can injure cardiac function and ultimately progress to heart failure [2]. One of the key pathophysiological features of HF is significant sympathetic nervous system activation, resulting in increased release of catecholamines, including isoprenaline. As a result, isoprenaline is frequently used to establish experimental animal models of HF, where its administration induces cardiac remodeling, myocardial hypertrophy, and systolic dysfunction [3]. In addition to isoprenaline-induced models, the transverse aortic constriction (TAC) model is widely employed to study heart failure [4]. This model mimics the pathophysiological changes associated with pressure overload by constricting the transverse aortic arch, which leads to an increased afterload on the left ventricle. The TAC model closely replicates the hemodynamic stress and remodeling processes observed in patients with conditions such as aortic stenosis or hypertension, ultimately causing left ventricular hypertrophy, diastolic and systolic dysfunction, and heart failure.

In the cardiac tissue of patients with HF, significant cardiomyocyte hypertrophy and cardiac fibrosis can be observed [5]. Because cardiomyocytes are considered terminally differentiated cells, they can undergo hypertrophy but not proliferation to compensate for injured cardiomyocytes. However, this can lead to increased oxygen and energy consumption and ultimately cause decompensation and promote cardiomyocyte death, which results in a vicious cycle. On the other hand, cardiac fibroblasts are able to proliferate and accelerate the formation of the extracellular matrix (ECM), causing augmented fibrosis and increased stiffness [6]. Cardiac fibrosis is another pathological feature of HF, making it difficult for dysfunctional hearts to contract smoothly or relax. Hence, relieving cardiomyocyte hypertrophy and cardiac fibrosis are core strategies for treating HF.

Currently, the treatment of heart failure is multifaceted, including pharmacological interventions, device-based therapies, and, in advanced cases, surgical approaches. The primary pharmacological treatments involve the use of angiotensin-converting enzyme inhibitors, angiotensin receptor blockers, beta-blockers, mineralocorticoid receptor antagonists, and, more recently, angiotensin receptor-neprilysin inhibitors to alleviate the progression of heart failure [7]. Diuretics are also widely used to manage symptoms related to fluid retention. While these medications provide symptomatic relief and improve survival in many patients, they primarily target hemodynamic factors and neurohormonal pathways. Despite their benefits, these treatments do not fully address the structural remodeling processes, such as cardiomyocyte hypertrophy and cardiac fibrosis, that drive disease progression.

Baicalin is one of the major active ingredients of the traditional Chinese herbal medicine Huang Qin (*Scutellaria baicalensis*) [8]. Huang Qin is used to treat patients with chest pain or cardiac discomfort in clinical work. By using liquid chromatography or mass spectrometry, the drug fingerprint spectrum of Huang Qin was revealed, and researchers reported that baicalin and baicalein are the two major pharmacologically active ingredients of Huang Qin [9]. Baicalin is an adduct of baicalein generated by the addition of glucuronate to baicalein. Interestingly, baicalein has been reported to alleviate several cardiac diseases, such as ischemia/reperfusion injury [10] and hypertension [11], supporting the cardioprotective effect of Huang Qin. Although baicalin is considered a metabolite of baicalein with limited biological activity, there is also evidence indicating that baicalin is able to relieve pressure overload-induced heart failure [12] and doxorubicin-induced cardiotoxicity [13]. However, whether baicalin is able to ameliorate isoprenaline-induced heart failure is still unknown, and the underlying mechanism(s) of the cardioprotective effect of baicalin is still not fully revealed.

In this work, we investigated the molecular mechanisms of baicalin in treating heart failure and explored a more effective therapeutic approach. We showed evidence that baicalin was able to ameliorate HF both in vivo and in vitro by reducing cardiomyocyte hypertrophy and relieving cardiac fibrosis. By using network pharmacology analysis and molecular docking, we showed that the cardioprotective effect of baicalin is dependent on the catalyzing subunit p85 of PI3K and that baicalin combined with PI3K p110 treatment had a stronger cardioprotective effect. In conclusion, we suggest that baicalin is also a useful cardioprotective agent via p85 of PI3K under HF and suggest the possibility of using baicalin to treat patients with HF in the clinic.

## 2. Materials and Methods

### 2.1. Animal Experimental Design

The animal experiments in this project were carried out with the approval of the Experimental Animal Centre of Huazhong University of Science and Technology (approval number: No. 2019005), ensuring compliance with ethical standards for the humane treatment of animals in research. Male wild-type C57BL6/J mice were housed in a specific pathogen-free (SPF) environment to minimize their exposure to infectious agents and ensure optimal health conditions throughout the study. These facilities were maintained under strict regulatory standards, with controlled temperature (22–26 °C) and humidity (40–60%) to replicate ideal living conditions. The mice were provided with a nutritionally balanced normal diet and clean drinking water, both of which were available ad libitum. A 12 h light/12 h dark cycle was implemented to mimic natural circadian rhythms, thereby minimizing stress and promoting physiological stability.

Isoprenaline model experiment: The isoproterenol model and treatments were used as described previously [12,14]. At the age of 8 weeks, forty mice were randomly divided into five groups: the PBS, ISO, ISO+Bai-L, ISO+Bai-H, and ISO+Pro groups, with *n* = 8 in each group. After 1 week of adaptation, 15 mg/kg isoprenaline or an equal volume of PBS was injected intraperitoneally each day for 3 weeks to establish the HF animal model. Baicalin was dissolved in 10% DMSO + 40% PEG300 + 5% Tween-80 + 45% saline and gavaged daily at dosages of 15 mg/kg or 25 mg/kg. Propranolol was used as a positive control at a dosage of 5 mg/kg/day. After 3 weeks, the animals were subjected to echocardiography and sacrificed by overdosage anesthetization. The PBS group: injection of PBS; the ISO group: injection of 15 mg/kg isoprenaline; the ISO+Bai-L group: injection of 15 mg/kg isoprenaline and 15 mg/kg baicalin; the ISO+Bai-H group: 15 mg/kg isoprenaline and injection of 25 mg/kg baicalin; the ISO+Pro group: injection of 15 mg/kg isoprenaline and 5 mg/kg propranolol.

Transverse aortic constriction (TAC) model experiment: The TAC model and medicines were used as described previously [12,15]. At the age of 8 weeks, forty mice were randomly divided into five groups: the sham, TAC, TAC+Bai-L, TAC+Bai-H, and TAC+Pro groups, with *n* = 8 in each group. The TAC operation was performed as described below. The mice were anesthetized with isoflurane and placed in a supine position. A midline incision was made along the neck and chest, followed by an incision at the second intercostal region at the left upper sternal border. A chest retractor was used to widen the incision, and the thymus, the aortic arch, and its branches were isolated and exposed. A 6-0 suture was placed around the aortic arch, and then the knot was made. A 26G cushion needle was positioned beside the aorta and tied tightly. After that, the needle was gently removed, and aortic stenosis with a lumen diameter of 0.4 mm was created, reducing the aortic diameter by 65–70%. The wound was then closed via 6–0 sutures, and postoperative care was provided. In the sham group, only a sham operation was performed, and the aorta was not tied. Baicalin was dissolved in 10% DMSO + 40% PEG300 + 5% Tween-80 + 45% saline and gavaged daily at dosages of 15 mg/kg or 25 mg/kg. Propranolol was used as a positive control at a dosage of 5 mg/kg/day. After 8 weeks, the animals were subjected to echocardiography and sacrificed by overdosage anesthetization. The sham group: sham operation; the TAC group: transverse aortic constriction; the TAC+Bai-L group: transverse aortic constriction and injection of 15 mg/kg baicalin; the TAC+Bai-H group: transverse aortic constriction and injection of 25 mg/kg baicalin; the TAC+Pro group: transverse aortic constriction and injection of 5 mg/kg propranolol.

Baicalin combined with LY294002 treatment model experiment: At the age of 8 weeks, thirty-two mice were randomly divided into four groups: the PBS, ISO, ISO+Bai, and ISO+Bai+LY294002 groups, with *n* = 8 in each group. The effects of baicalin and isoproterenol were consistent with the results of the isoprenaline model experiment. LY294002 was dissolved in 10% DMSO + 40% PEG300 + 5% Tween-80 + 45% saline and injected intraperitoneally daily at a dosage of 25 mg/kg [16]. After 8 weeks, the animals were subjected to echocardiography and sacrificed by overdosage anesthetization. The PBS group: injection of PBS; the ISO group: injection of 15 mg/kg isoprenaline; the ISO+Bai group: injection of 15 mg/kg isoprenaline and 25 mg/kg baicalin; the ISO+Bai+LY294002 group: injection of 15 mg/kg isoprenaline, 25 mg/kg baicalin and 25 mg/kg LY294002.

### 2.2. Primary Neonatal Rat Cardiomyocytes (NRCMs) or Cardiac Fibroblasts (NRCFs) Culture

Using the methods previously reported [17], NRCMs and NRCFs were separated via the enzymatic digestion of type II collagenase, trypsin, and benzonase. After the heart was harvested and arterial tissue was removed from 1–4-day-old neonatal rats, the ventricles were cut into small pieces and digested with an enzyme mixture at 37 °C for 40 min with gentle shaking. After thorough digestion, the cell suspension was filtered through a cell filter (BIOFIL, CSS013070, Guangzhou, China) and centrifuged to harvest the cell pellet. The red blood cells in the cell pellet were lysed via red-blood-cell lysis. The cell pellet was subsequently resuspended in DMEM/F12 culture medium supplemented with a 1% penicillin/streptomycin mixture and 10% fetal bovine serum. Fibroblasts and CMs were separated by differential attachment to a cell culture plate. The culture medium of cardiomyocytes was further supplemented with 5-BrdU to suppress the growth of contaminating fibroblasts.

### 2.3. Antibodies and Agents

The following antibodies were used in this work: PI3Kphos607tyr (Absin, AP11, Shanghai, China), PI3K (ABclonal, A4992, Wuhan, China), Akt phos473ser (CST, 9271, Danvers, MA, USA), Vimentin (Boster, BM1035, Wuhan, China), aSMA (Boster, BM0002 Wuhan, China), NPPA (ABclonal, A22075, Wuhan, China), NPPB (ABclonal, A23996, Wuhan, China), MYH7 (ABclonal, A24664, Wuhan, China), fibronectin (ABclonal, A16678, Wuhan, China), and HRP-conjugated goat anti-Rabbit IgG (ABclonal, AS014, Wuhan, China). The following agents were used in this work: baicalin (Aladdin, B110211, Shanghai, China), isoprenaline (Sigma-Aldrich, I5627, St. Louis, MO, USA), type II collagenase (Sigma-Aldrich, LS004176, St. Louis, MO, USA), Angiotensin II (MCE, HY-13948, Monmouth Junction, NJ, USA), trypsin (BioFROXX, 1004GR025, Offenbach am Main, Germany), antibody dilution buffer (Abbkine, BMU103, Wuhan, China), a supersensitive ECL kit (Abbkine, BMU102, Wuhan, China), 740-Y-P (MCE, HYP-0175, Monmouth Junction, NJ, USA), LY294002 (MCE, HY-10108, Monmouth Junction, NJ, USA), propranolol (MCE, HY-B0573B, Monmouth Junction, NJ, USA), red-blood-cell lysis (Servicebio, G2015, Wuhan, China), penicillin/streptomycin mixture (Servicebio, G4003, Wuhan, China), fetal bovine serum (Yeasan, 40131ES76, Shanghai, China), DMEM/F12 culture medium (Servicebio, G4610, Wuhan, China), DAB kit (G1212, Servicebio, Wuhan, China), proteinase inhibitor and phosphatase inhibitor cocktail (Yeasan, 20123ES and 20109ES, Shanghai, China), BCA assay kit (Yeasan, 20201ES, Shanghai, China), skim milk (Servicebio, GC310001, Wuhan, China).

### 2.4. Echocardiography Examination

Animals were examined via Vevo®1100 (Fujifilm VisualSonics, Toronto, ON, Canada) according to previous reports [18]. Briefly, the animals were anesthetized with isoflurane, and the heart rate was monitored via ECG. The heart rate was controlled between 550 and 600 bpm by adjusting the concentration of isoflurane. A standard parasternal long-axis section of the heart was captured, and the sampling line in M-mode was placed next to the papillary muscle. The ejection fraction (EF%), ventricular wall thickness and left ventricular mass were subsequently calculated via LV analysis via Vevo®1100 software.

### 2.5. Hematoxylin–Eosin (H&E) Staining and Sirius Red Staining

Using the methods previously reported [19]. For hematoxylin and eosin (H&E) staining and Sirius Red staining, a commercial kit was used according to the manufacturer’s protocol (Servicebio, G1076 and G1078, Wuhan, China). Briefly, fresh tissues were fixed in 4% paraformaldehyde for at least 24 h, trimmed, and placed into dehydration cassettes with appropriate labeling. The tissues underwent a graded alcohol dehydration process, followed by clearing with xylene and embedding in paraffin wax. After embedding, 4 μm-thick sections were cut using a microtome and floated on 40 °C warm water to flatten. The sections were mounted on slides, baked at 60 °C to remove residual water and wax, and stored at room temperature until further use.

For staining, the paraffin-embedded sections were dewaxed in xylene and rehydrated through a graded alcohol series. For H&E staining, sections were stained in hematoxylin solution to visualize nuclei, rinsed, and counterstained with eosin for cytoplasm and extracellular matrix. For Sirius Red staining, sections were incubated in buffer A for 2 min and buffer B for 30 min to detect collagen fibers. Finally, the sections were dehydrated, cleared in xylene, and mounted with neutral resin for microscopic examination. Each step was carefully performed to preserve tissue integrity and ensure high-quality staining results. Collagen deposition was evaluated to quantify the extent of cardiac fibrosis by calculating the fibrotic area relative to the total tissue area.

For each tissue section, three images were obtained for each type of staining to ensure representative sampling of the histological characteristics. For each animal, only the heart was sampled for histological examination. Each experimental group consisted of *n* = 6 animals, and tissue sections were consistently prepared and stained to maintain uniformity across the study.

### 2.6. Immunohistochemistry and Immunofluorescence Staining

All these procedures were conducted according to previous reports [18,20]. Briefly, for paraffin-embedded tissue sections, fresh tissues were fixed in 4% paraformaldehyde for 24 h, processed through an alcohol gradient for dehydration, cleared in xylene, and embedded in paraffin. Tissue blocks were sectioned into 4 μm slices using a microtome, floated on warm water (40 °C) to flatten the sections, transferred to glass slides, and dried in a 60 °C oven.

For immunohistochemistry (IHC), sections were first dewaxed in xylene and rehydrated through a graded ethanol series. Non-specific antigen-antibody binding was blocked by incubating the sections in 5% BSA and 10% goat serum in PBS at room temperature for 30 min. The sections were then incubated overnight at 4 °C with primary antibodies diluted according to the manufacturer’s instructions. After incubation, excess primary antibodies were removed by washing the sections three times with PBS. HRP-conjugated secondary antibodies (1:200 dilution) were applied and incubated at room temperature for 2 h. Signals were visualized using a DAB detection kit (Servicebio, G1212, Wuhan, China) according to the manufacturer’s protocol. After staining, the sections were dehydrated, cleared in xylene, and mounted with resin for further microscopic analysis under a bright-field microscope.

For immunofluorescence staining, cells or tissues were fixed with 4% paraformaldehyde and permeabilized with 0.1% saponin in PBS. Primary antibodies were applied at manufacturer-recommended dilutions and incubated overnight at 4 °C. After three washes with PBS, Cy3- or Alexa Fluor 488-conjugated secondary antibodies were added and incubated at room temperature for 1 h in the dark. Excess secondary antibodies were removed by washing with PBS. Fluorescence images were captured using a Nikon laser confocal microscope (Nikon, Tokyo, Japan) or a Zeiss bright-field microscope (Zeiss, Jena, Germany).

For each tissue section, three images were obtained for each type of staining to ensure representative sampling of the histological characteristics. For each animal, only the heart was sampled for histological examination. Each experimental group consisted of *n* = 6 animals, and tissue sections were consistently prepared and stained to maintain uniformity across the study.

### 2.7. Protein Extraction and Western Blot

Cellular protein or tissue protein was extracted with RIPA lysis buffer supplemented with proteinase inhibitor and phosphatase inhibitor cocktail. The mixture was incubated on ice for 15 min and then vortexed every 5 min. The tissue was minced by a tissue lysis machine with a 3 mm steel ball at 4 degrees centigrade. Then, the lysate was centrifuged at 16000× *g* for 15 min to harvest the supernatant. The protein concentration was quantified with a BCA assay kit. After the protein concentration was adjusted, the sample was boiled with loading buffer. Immunoblotting was carried out according to previous methods [21]. The proteins were transferred to a PVDF membrane. After blocking with 5% skim milk for 1 h, The membrane was incubated with specific primary antibodies at a dilution of 1:1000. This was followed by incubation with secondary antibodies diluted 1:5000. The bands were visualized by enhanced chemiluminescence (ECL) and analyzed with ImageJ.

### 2.8. Network Pharmacology Analysis, Target Identification, and Molecular Docking

The SMILES of baicalin was searched in the PubChem database, and the SMILES was uploaded into PharmMapper [22], SuperPred, and STITCH. After the molecular structure of baicalin was downloaded, the molecule was docked to PI3K p85 (8DCP in PDB) by Schrödinger-2022-2. All target genes were normalized and imported into the UniProt database to obtain the UniProt ID. After the UniProt ID was summarized, all the duplicates were removed. The keywords “myocardial injury” and “fibrosis” were input into GeneCards to search for all target genes. All target genes were normalized and imported into the UniProt database to obtain the UniProt ID. After the UniProt ID was summarized, all the duplicates were removed. A Venn diagram was drawn to show the intersection targets of myocardial injury and fibrosis with baicalin. SRTING is an interactive gene database search tool that can obtain protein–protein interaction (PPI) information. The common targets were entered into STRING11.0, *Homo sapiens* was a limited organism, and the minimum required interaction score was set to the highest confidence (0.900). Cytoscape 3.7 was used to construct a common target PPI network diagram. The node size and color were adjusted according to the degree in the diagram. Finally, the DAVID (https://david.ncifcrf.gov/, accessed on 30 May 2023) database was used to collect GO analysis and KEGG data [23]. GO analysis is used to screen biological processes (BP), cellular components (CC), and molecular functions (MF) [24]. KEGG enrichment analysis revealed important signaling pathways involved in biological processes. The GO and KEGG data were subsequently uploaded to the Bioinformatics (http://www.bioinformatics.com.cn/, accessed on 30 May 2023) platform for visual analysis. 

### 2.9. Statistical Analysis

Statistical analysis was performed using GraphPad Prism 8.0.2 software (GraphPad Software, Inc., La Jolla, CA, USA). Prior to selecting an appropriate ANOVA, the normality of data distribution was assessed using the Shapiro–Wilk test. For data with a normal distribution, comparisons between two groups were conducted using an unpaired Student’s *t*-test, while one-way ANOVA followed by Tukey’s post hoc test was employed for multiple group comparisons. For non-normally distributed data, non-parametric ANOVA was applied. The statistical test used, exact *p*-values, and the number of replicates (*n*) are indicated in the individual figure legends. Values of * *p* < 0.05, ** *p* < 0.01, *** *p* < 0.001, and **** *p* < 0.0001 were considered statistically significant between groups. Every possible comparison between the study groups was considered.

## 3. Results

### 3.1. Baicalin Can Reverse Cardiomyocyte Hypertrophy and Heart Failure

**Figure 1:**
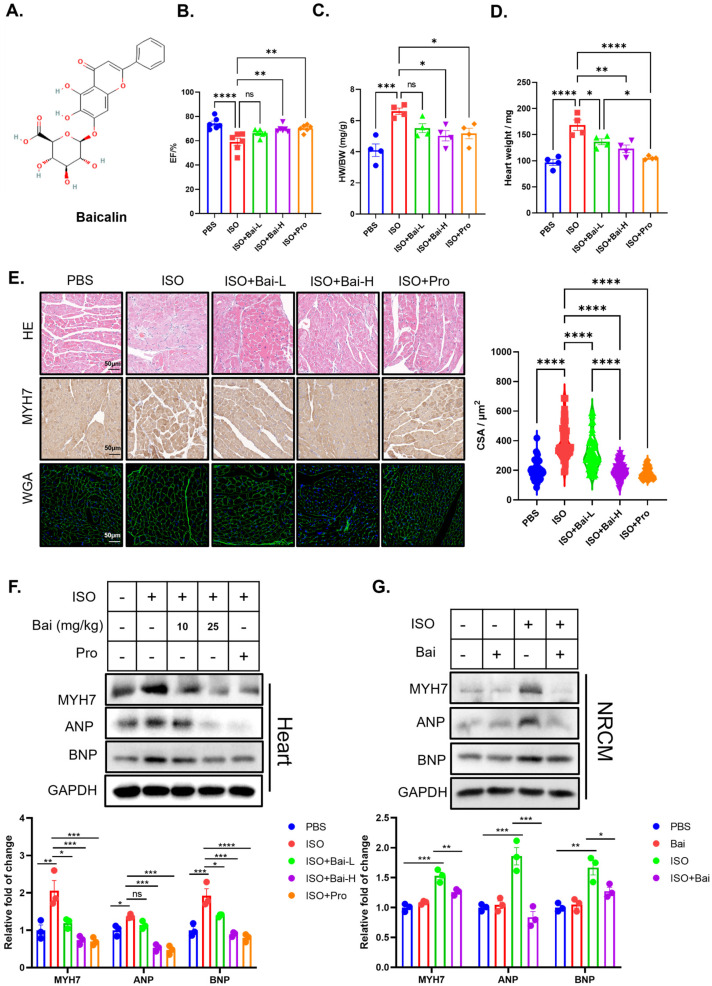
Baicalin ameliorates isoprenaline-induced heart failure and reduces cardiomyocyte hypertrophy. (**A**) Chemical construction of baicalin. (**B**) Left ventricular ejection fraction of the mice in the five groups (*n* = 6). (**C**) Ratios of heart weight to body weight to body weight of the mice in the five groups (*n* = 4). (**D**) Heart weights of the mice in the five groups (*n* = 4). (**E**) Representative images of H&E staining, immunohistochemical staining of MYH7, and WGA staining of paraformaldehyde-fixed cardiac tissue (*n* = 50). (**F**) Representative western blot image of markers of hypertrophy (including MYH7, ANP, and BNP) and GAPDH in the heart tissue of the five groups (*n* = 3). (**G**) Representative western blot image of markers of hypertrophy (including MYH7, ANP, and BNP) and GAPDH in NRCMs treated with isoprenaline and baicalin (*n* = 3), isoprenaline (ISO), low doses of baicalin (Bai-L), high doses of baicalin (Bai-H), propranolol (Pro), baicalin (Bai). (* *p* < 0.05, ** *p* < 0.01, *** *p* < 0.001, **** *p* < 0.0001, ns, no significant difference; all the significant data were analyzed via ANOVA).

To fully reveal the protective effect of baicalin (Bai) (Figure 1A) on the heart, we first established an isoprenaline (ISO)-induced heart failure animal model in male C57Bl6 mice, and since isoprenaline is a classical beta receptor agonist, we also used propranolol (Pro) as a positive control to evaluate the effect of baicalin. Similar to the literature [3], isoprenaline significantly deteriorated left ventricular contraction ability and caused cardiac hypertrophy, as evidenced by a reduced left ventricular ejection fraction (Figure 1B) and increased heart weight-to-body weight ratio (Figure 1C) and left ventricular mass (Figure 1D). Surprisingly, treating mice with baicalin effectively preserved heart function and ameliorated cardiac hypertrophy, as did propranolol (Figure 1B–D).

We further observed pathological changes in mouse cardiac tissue and found that isoprenaline caused significant inflammatory cell infiltration via H&E staining and that cardiomyocytes expressed higher levels of Myosin Heavy Chain 7 (MYH7), a biomarker of hypertrophied myocardium. Wheat germ agglutinin (WGA) staining revealed a larger cross-sectional area of cardiomyocytes (Figure 1E). All these isoprenaline-induced pathological features were reversed by baicalin treatment (Figure 1E). We also measured the protein levels of MYH7, atrial natriuretic peptide (ANP), and brain natriuretic peptide (BNP), which are established biomarkers for cardiac hypertrophy and heart failure, in mouse cardiac tissue and found significant increases in these biomarkers, which were reversed by baicalin treatment (Figure 1F). These data suggested that baicalin treatment effectively ameliorated isoprenaline-induced heart failure in vivo.

Next, we wanted to test whether baicalin could exhibit cardioprotective effects in vitro. We confirmed that baicalin could also reduce isoprenaline-induced hypertrophy in neonatal rat cardiomyocytes (NRCMs) (Figure 1G) and reverse the expression levels of biomarkers of heart failure and hypertrophy. In conclusion, these data suggested that baicalin was able to ameliorate isoprenaline-induced cardiomyocyte injury both in vivo and in vitro.

**Figure 2:**
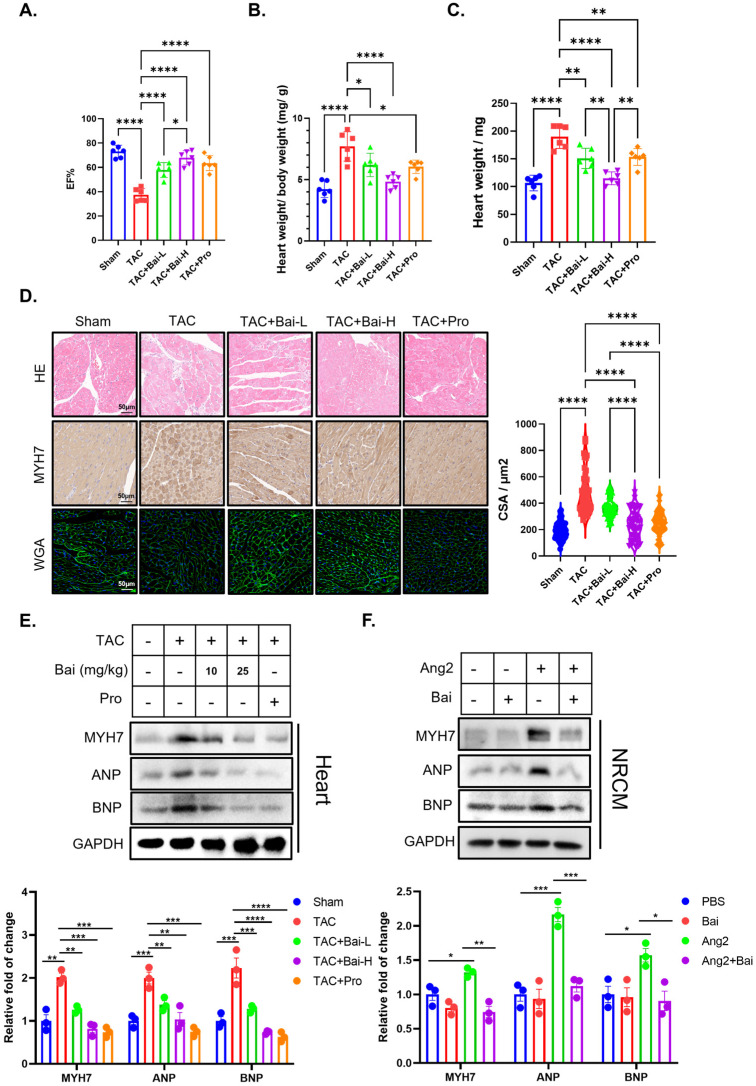
Baicalin ameliorates transverse aortic constriction-induced heart failure and reduces cardiomyocyte hypertrophy. (**A**) The left ventricular ejection fraction of mice in four groups (*n* = 6). (**B**) the ratio of heart weight to body weight to body weight of mice in four groups (*n* = 6). (**C**) heart weight of mice in four groups (*n* = 6). (**D**) representative image of H&E staining, immunohistochemistry staining of MYH7, and WGA staining of paraformaldehyde fixed cardiac tissue (*n* = 50). (**E**) Representative western blot image of markers of hypertrophy (including MYH7, ANP, BNP) and GAPDH in heart tissue of five groups (*n* = 3). (**F**) Representative western blot image of markers of hypertrophy (including MYH7, ANP, and BNP) and GAPDH in NRCMs treated with Angiotensin II and baicalin (*n* = 3). transverse aortic constriction (TAC), low doses of baicalin (Bai-L), high doses of baicalin (Bai-H), propranolol (Pro), baicalin (Bai), and primary neonatal rat cardiomyocytes (NRCMs). (* *p* < 0.05, ** *p* < 0.01, *** *p* < 0.001, **** *p* < 0.0001, all the data significance was analyzed by ANOVA).

The aortic arch constriction model simulates hypertension-induced HF, augmenting the left ventricular afterload. Consistent with the heart failure model induced by isoproterenol, baicalin also had significant protective effects on the TAC model (Figure 2).

### 3.2. Baicalin Targets the PI3K Signaling Pathway as Revealed by Integrated Network Pharmacology Analysis

**Figure 3:**
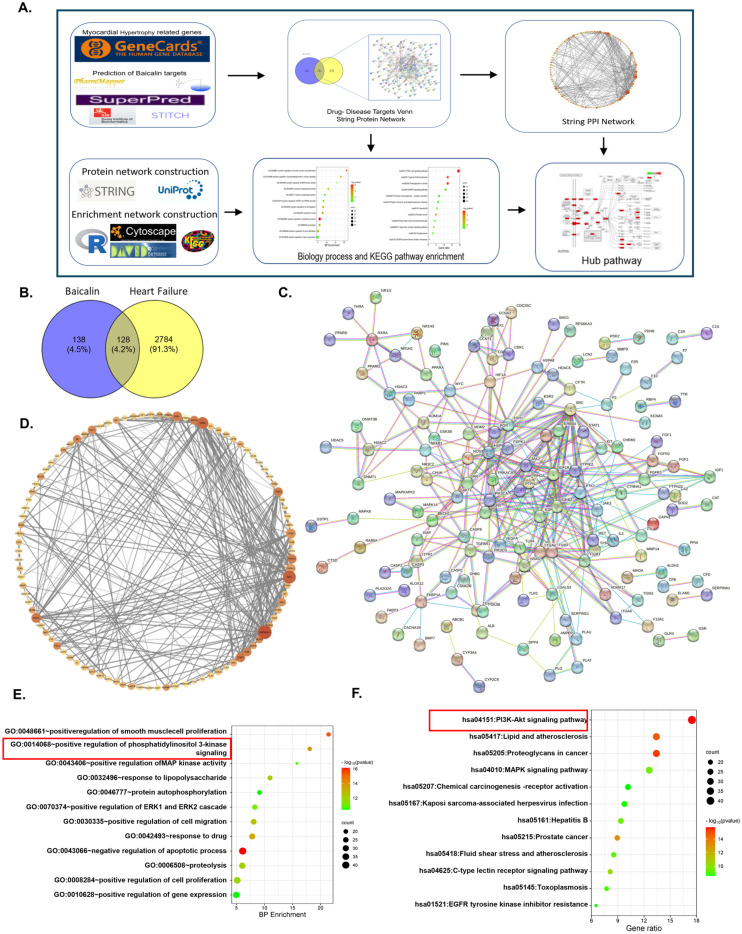
Baicalin targets the PI3K signaling pathway. (**A**) Workflow of the integrated network pharmacology analysis. (**B**) Overlapping genes of potential baicalin target genes and heart failure-related genes. (**C**) Protein–protein interactions of 128 overlapping genes in B analyzed via STRING. (**D**) PPIs were analyzed by Cystoscope. (**E**,**F**) Gene Ontology (GO) (**E**) and Kyoto Encyclopedia of Genes and Genomes (KEGG) (**F**) enrichment of overlapping genes between baicalin and heart failure.

**Figure 4:**
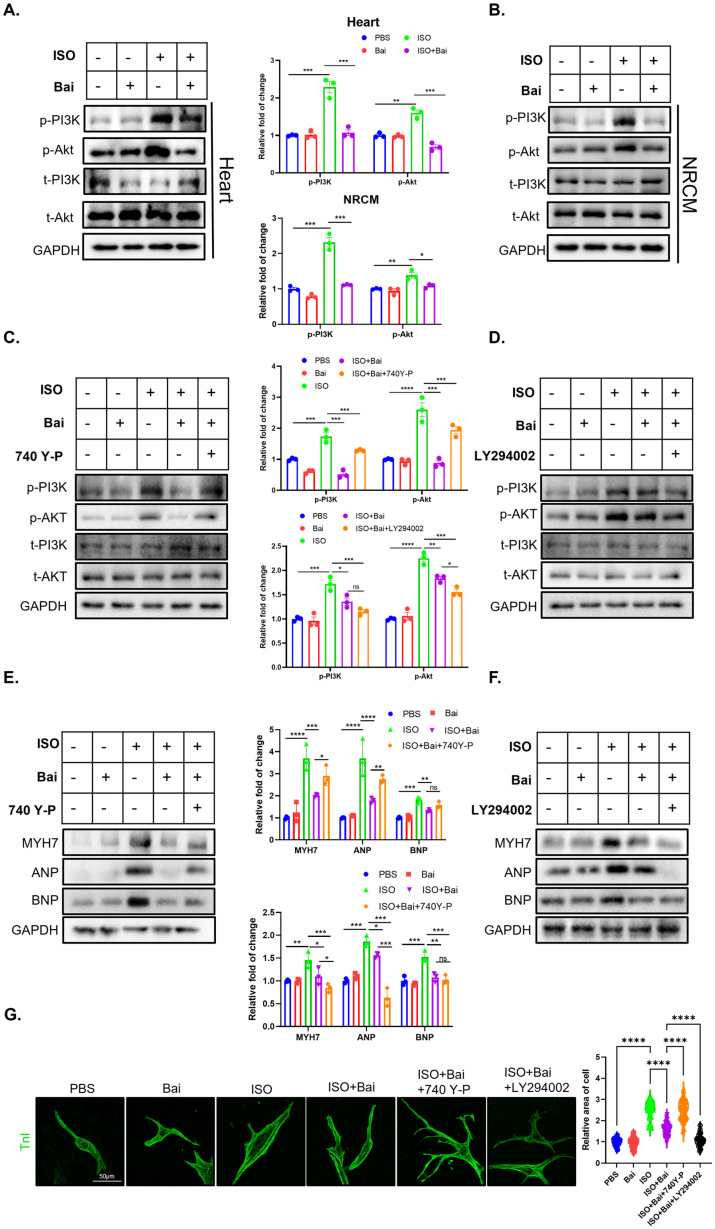
The antihypertrophic effect of baicalin was dependent on the activity of PI3K. (**A**) Representative western blot images of markers of the PI3K–Akt signaling pathway and GAPDH in the heart tissues of the four groups (*n* = 3). (**B**) Representative western blot image of markers of the PI3K–Akt signaling pathway and GAPDH in NRCMs treated with isoprenaline and baicalin (*n* = 3). (**C**,**D**) Representative western blot image of the PI3K–Akt signaling pathway in NRCMs treated with isoprenaline, baicalin or 740-Y-P, an agonist of PI3K (**C**), and LY294002, an antagonist of PI3K (**D**) (*n* = 3). (**E**,**F**) Representative western blot image of markers of hypertrophy (including MYH7, ANP, and BNP) in NRCMs treated with isoprenaline, baicalin or 740-Y-P, an agonist of PI3K (**E**), and LY294002, an antagonist of PI3K (**F**) (*n* = 3). (**G**) Representative confocal image of NRCMs treated with baicalin, isoprenaline, or a PI3K antagonist or agonist. NRCMs were stained with troponin I (*n* = 50), isoprenaline (ISO), baicalin (Bai), and primary neonatal rat cardiomyocytes (NRCMs). (* *p* < 0.05, ** *p* < 0.01, *** *p* < 0.001, **** *p* < 0.0001, ns, no significant difference; all the significant data were analyzed via ANOVA).

One of the long-standing problems in investigating the pharmacological mechanism(s) of active TCM ingredients is that the exact molecular target(s) is difficult to confirm. Recently, owing to the development of several TCM databases and bioinformatic databases, researchers have analyzed the potential molecular targets of active TCM ingredients. Hence, we integrated several databases (Figure 3A) and identified 128 overlapping genes between potential targets of baicalin (Figure 3B, blue circle) and genes anticipated to be involved in the development of heart failure (Figure 3B, yellow circle). We then constructed a protein–protein interaction (PPI) network with STRING (Figure 3C) and Cystoscope (Figure 3D), and the PPI network included several hub genes, including PIK3CG, Akt, HSP90AA, ITGB, and Src. These highlighted genes are well-reported in the field of heart failure. We also analyzed the Gene Ontology (GO) and Kyoto Encyclopedia of Genes and Genomes (KEGG) enrichment of these genes and detected significant enrichment of the PI3K–Akt signaling pathway (Figure 3E,F). Hence, network pharmacology analysis indicated that the PI3K–Akt signaling pathway may be the core molecular target of baicalin. Consistent with the network pharmacology prediction, we found that the PI3K–Akt signaling pathway was significantly activated by isoprenaline stimulation and reversed by baicalin treatment both in vivo (Figure 4A) and in vitro (Figure 4B).

To further confirm that PI3K is indispensable for the cardioprotective effect of bai, we applied a PI3K agonist and antagonist (Figure 4C–G) and found that the PI3K agonist 740 Y-P [25] attenuated the antihypertrophic effect of bai, as evidenced by the preserved expression of MYH7, ANP, and BNP after 740 Y-P treatment (Figure 4E). In contrast, LY294002 [26] exhibited cardioprotective effects similar to those of bai (Figure 4F). Immunofluorescence staining of the cross-sectional area of NRCMs also yielded parallel western blot results (Figure 4G). Hence, these data suggest that bai inhibits p85 activity to exert a protective effect on cardiomyocytes by inhibiting PI3K activity.

### 3.3. Baicalin Could Ameliorate Cardiac Fibrosis

**Figure 5:**
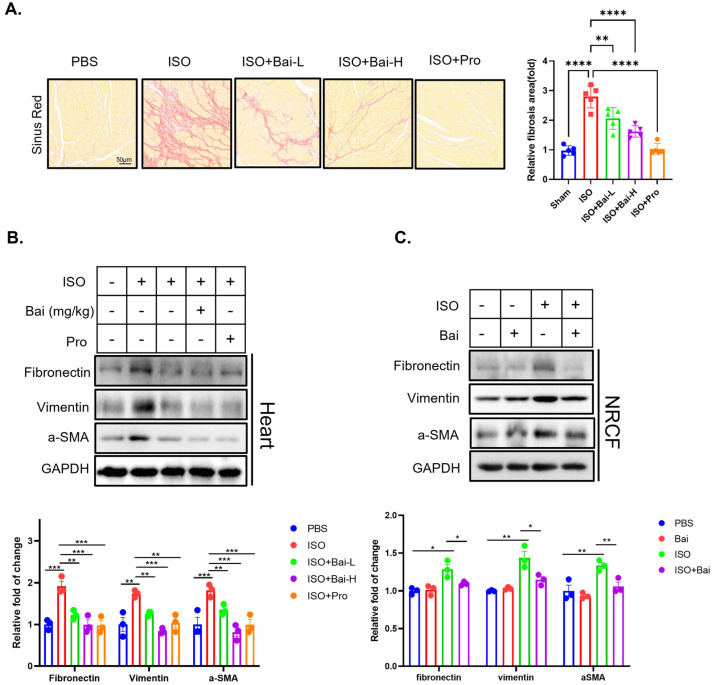
The antifibrotic effect of baicalin was dependent on the activity of PI3K. (**A**) Representative image of Sirius Red staining of paraformaldehyde-fixed cardiac tissue (*n* = 5). (**B**) Representative western blot images of markers of fibrosis (including fibronectin, vimentin, and a-SMA) and GAPDH in heart tissue from the five groups (*n* = 3). (**C**) Representative western blot image of markers of fibrosis (including fibronectin, vimentin, and a-SMA) and GAPDH in NRCFs treated with isoprenaline, baicalin, and propranolol (*n* = 3), isoprenaline (ISO), low doses of baicalin (Bai-L), high doses of baicalin (Bai-H), propranolol (Pro), baicalin (Bai), and primary neonatal rat cardiac fibroblasts (NRCFs). (* *p* < 0.05, ** *p* < 0.01, *** *p* < 0.001, **** *p* < 0.0001; all the significant data were analyzed via ANOVA).

As another outstanding pathological feature of heart failure, enhanced cardiac fibrosis contributes to augmented myocardial stiffness and reduced diastolic function. Sirius Red staining is valuable for assessing cardiac fibrosis. It specifically binds to collagen fibers, with the intensity of staining reflecting the amount of fibrosis, making it an excellent method for visualizing and quantifying fibrosis in heart tissue. Isoprenaline stimulation caused significant cardiac fibrosis, as evidenced by the significant red-stained signal of the sinus (Figure 5A), which is consistent with previous reports [27]. We observed that baicalin treatment also ameliorated isoprenaline-induced cardiac fibrosis (Figure 5A). We further measured biomarkers of fibrosis, such as fibronectin, vimentin, and a-SMA, and found that baicalin downregulated the expression of these biomarkers of fibrosis both in vivo (Figure 5B) and in vitro (Figure 5C).

**Figure 6:**
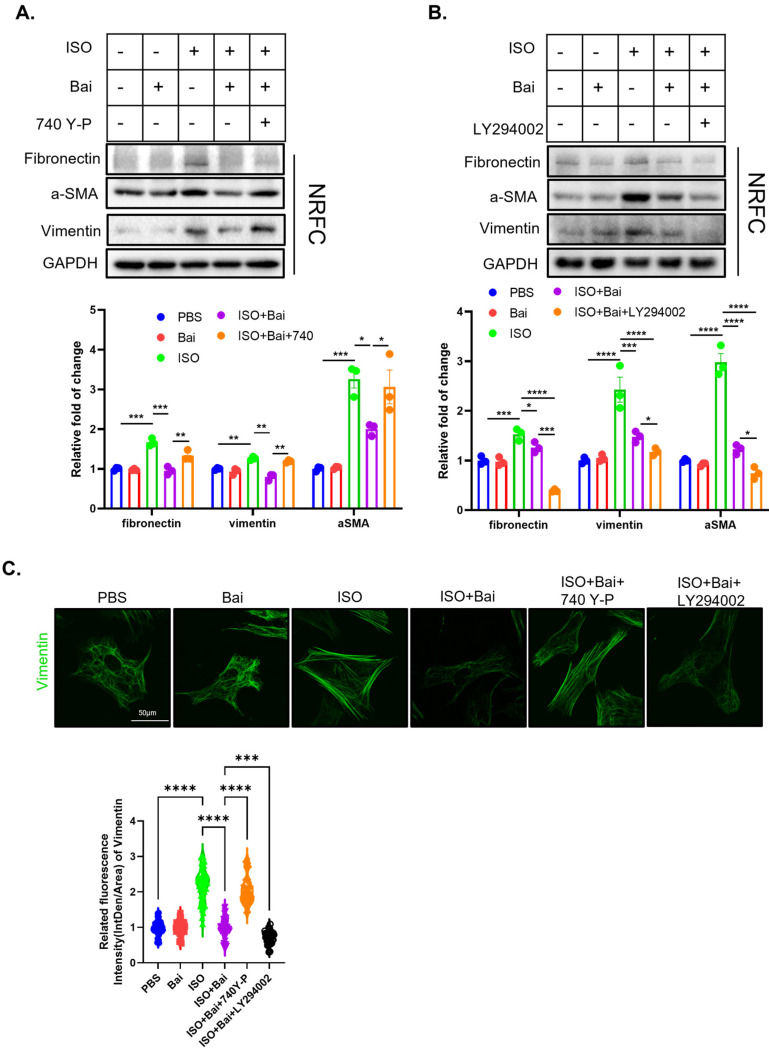
The antifibrotic effect of baicalin was dependent on the activity of PI3K. (**A**,**B**) Representative western blot image of markers of fibrosis (fibronectin, vimentin, and a-SMA) in NRCFs treated with isoprenaline, baicalin, or 740-Y-P, an agonist of PI3K (**A**), and LY294002, an antagonist of PI3K (**B**) (*n* = 3). (**C**) Representative confocal image of NRCFs treated with baicalin, isoprenaline, or a PI3K antagonist or agonist (*n* = 50). NRCFs were stained with vimentin and DAPI. isoprenaline (ISO), baicalin (Bai), and primary neonatal rat cardiac fibroblasts (NRCFs). (* *p* < 0.05, ** *p* < 0.01, *** *p* < 0.001, **** *p* < 0.0001; all the significant data were analyzed via ANOVA).

Similar to the cardiomyocyte-protective effect of baicalin on NRCMs, the antifibrotic effect of baicalin on NRCFs was also related to the inhibition of PI3K activity (Figure 6A–C), as evidenced by the decreased expression of fibrotic biomarkers after the administration of the PI3K antagonist (Figure 6A) and increased expression of these biomarkers after the application of the PI3K agonist (Figure 6B). The immunofluorescence results revealed findings similar to those observed for vimentin via Western blot analysis. (Figure 5C).

Hence, we concluded that baicalin also has antifibrotic effects via the PI3K pathway under cardiac fibrosis.

### 3.4. The Combination Treatment of Baicalin and Inhibitor of PI3K p110 Exhibited Enhanced Cardioprotective Efficacy

**Figure 7:**
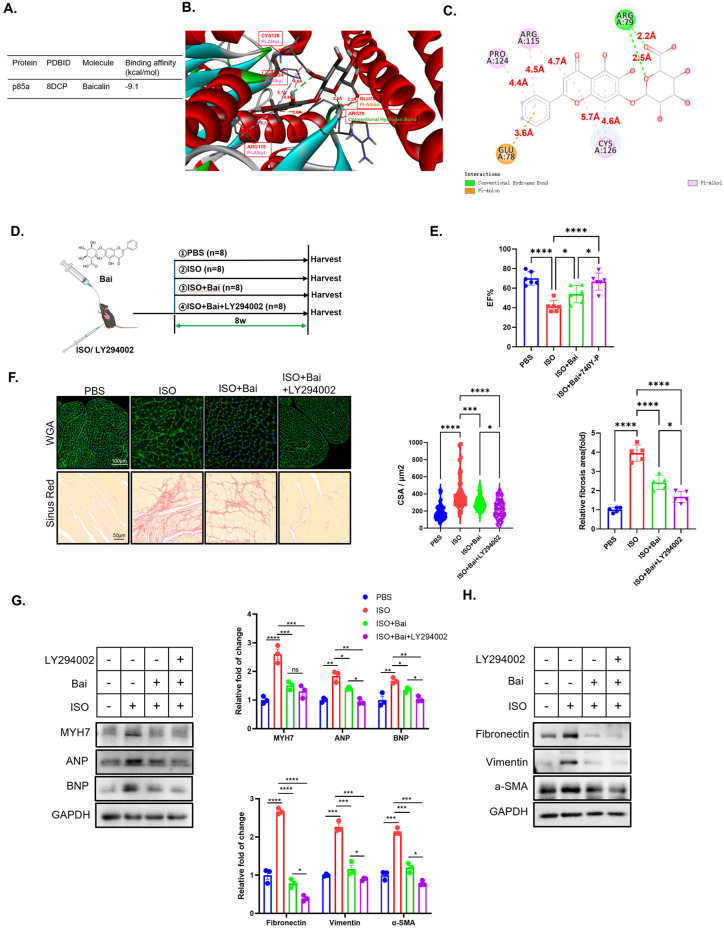
Combination treatment with baicalin and an inhibitor of PI3K p110 resulted in enhanced cardioprotection efficacy. (**A**) binding affinity between baicalin and p85a according to molecular docking. (**B**) The 3D structure of interaction between five amino acid residues and baicalin. (**C**) The 2D molecular interaction illustration of interaction between baicalin and five key amino acid residues. (**D**) Baicalin combined with LY294002 was used to treat the animal model (*n* = 3). (**E**) Left ventricular ejection fraction of the mice (*n* = 6). (**F**) Representative image of Sirius Red and WGA staining of paraformaldehyde-fixed cardiac tissue(*n* = 50 for WGA, *n* = 5 for Sirius Red). (**G**) Representative western blot image of markers of hypertrophy (including MYH7, ANP, and BNP) and GAPDH in the heart tissue of the four groups (*n* = 3). (**H**) Representative western blot image of markers of fibrosis (including fibronectin, vimentin and a-SMA) and GAPDH in the heart tissue of the four groups (*n* = 3), isoprenaline (ISO), baicalin (Bai). (* *p* < 0.05, ** *p* < 0.01, *** *p* < 0.001, **** *p* < 0.0001, ns, no significant difference; all the significant data were analyzed via ANOVA).

PI3K is a large complex comprising four catalytic subunits and two regulatory subunits [28]. Since network pharmacology analysis indicates that PI3K may be the target of baicalin, we first downloaded the structures of the subunits of PI3K from the PDB and virtually docked baicalin to the molecular structures by Schrödinger. We found that the p85a regulatory subunit had the highest affinity among all the subunits of PI3K (Figure 7A) and that baicalin could interact with the Glu78, Arg79, Arg115, Pro124, and Cys126 of p85 (Figure 7B,C). Given that baicalin mediates its cardioprotective effects through inhibition of the p85α subunit of PI3K, the question of whether the combination of baicalin with an inhibitor targeting the p110 subunit of PI3K could result in an enhanced cardioprotective effect is raised. In our study on baicalin treatment for isoprenaline-induced HF, we specifically supplemented the treatment with LY294002, a selective PI3K inhibitor (Figure 7D). The additional administration of LY294002 further increased the ejection fraction (Figure 7E). Furthermore, the morphological data indicated that the additional administration of LY294002 further enhanced the effects of baicalin on the red-stained signals of the Sirius Red and cardiomyocyte areas (Figure 7F). Western blot analysis also revealed that the combination of baicalin and LY294002 further reduced the expression of markers of cardiomyocyte hypertrophy (Figure 7G) and myocardial fibrosis (Figure 7H).

## 4. Discussion

In this study, we gave in vivo and in vitro evidence that baicalin can ameliorate two major pathological features of heart failure, cardiomyocyte hypertrophy and cardiac fibrosis, via the inhibition of PI3K activity. By applying integrated network pharmacology analysis, we determined that the PI3K signaling pathway was responsive to baicalin treatment, and we further used the predicted binding site in p85 to baicalin to support the idea that baicalin binds with p85a. In addition, by implementing a specific agonist and antagonist of PI3K together with baicalin, we further demonstrated that baicalin indeed inhibited the activity of PI3K to display cardioprotective effects, as biomarkers for hypertrophy and fibrosis were again elevated after treating NRCMs or NRCFs with a PI3K agonist. Finally, baicalin combined with LY294002, an inhibitor of PI3K p110, had a stronger cardioprotective effect on ISO-induced HF. Therefore, baicalin combined with LY294002 may be a more effective treatment strategy.

As the active component of the TCM herb Huang Qin, the pharmacological effect of baicalin has drawn attention in the field of phytochemicals. Baicalin has been proven to be effective in treating various diseases, including various types of cancers [29], inflammatory diseases [30], autoimmune diseases [31], and cardiovascular diseases [32], suggesting the value of baicalin in basic pharmacology research and its potential clinical effects. In the field of cardiovascular disease research, the application of baicalin has focused primarily on pressure overload-induced heart failure [12,33], ischemia/reperfusion injury [34,35] or cardiac toxicity [13,36]. However, as one of the important animal models for investigating the mechanism of HF, little attention has been given to isoprenaline-induced heart failure, and our work addresses the pharmacological protective effect of baicalin in treating cardiac diseases. Therefore, although baicalin also had significant cardioprotective effects on the TAC model, we selected isoproterenol-induced HF as the study model. We found that baicalin had a protective effect similar to that of the classical beta blocker propranolol. As isoprenaline administration is an important animal model for establishing HFs, various studies have revealed that the pathological features of isoprenaline administration include significant fibrosis, cardiomyocyte apoptosis and hypertrophy [37,38,39]. In our study, baicalin alleviated these pathological features, indicating that the treatment effect of baicalin is worthy of future investigation.

As one of the most important signaling pathways in eukaryotes, the PI3K signaling pathway participates in regulating various life processes, such as proliferation [40], differentiation [41], apoptosis [42], and glucose uptake [43]. PI3Ks are categorized into three types, the most well-studied of which is type I PI3Ks, which are heterodimers composed of a regulatory subunit and a catalytic subunit [44]. In our work, integrated network pharmacology analysis indicated that PI3K is the major target of baicalin, and we compared the calculated binding affinity between baicalin and different subunits of PI3K and found that p85a displayed the highest binding affinity. Our work is consistent with previous reports, indicating that PI3K is indeed a target of baicalin. In these works, baicalin was shown to inhibit the PI3K signaling pathway to treat osteosarcoma [45], glaucoma [46], spinal cord injury [47], neuroinflammation [48], and pulmonary hypertension [49]. Our work provides insight into the inhibitory effect of baicalin on PI3K in the cardiovascular system. By molecular docking prediction, we found that the 78th, 79th, 115th, 124th and 126th amino acid residues interacted with baicalin. Interestingly, the SH3 domain of p85a is located at the 3rd–79th residues, which indicates that baicalin may interfere with the conformation of SH3 to inhibit the activity of p85a.

PI3K can further phosphorylate Akt and promote cardiomyocyte hypertrophy. As a downstream target of PI3K, we found that Akt was also phosphorylated by isoprenaline treatment and was dephosphorylated by baicalin treatment. This finding is consistent with the knowledge that overactivated Akt can promote pathological hypertrophy and heart failure [50]. An important strategy to alleviate the workload of the myocardium is to reduce hypertrophy, since hypertrophied cardiomyocytes are in greater demand of energy supplementation, which results in metabolic remodeling of the failing heart. Recently, targeted metabolomics research revealed that the myocardium of patients with HF contains fewer long-chain acylcarnitine and more ketones [51], supporting the theory that the HF heart converts its metabolic substrate from fatty acids to ketones or glucose [52]. In our work, baicalin dose-dependently reduced the degree of hypertrophy in cardiomyocytes and preserved the ratio of heart weight to body weight. This finding suggests that alleviating hypertrophy can reduce energy consumption and hence improve heart function.

Insulin resistance is a well-established condition that can contribute to the development of diabetic cardiomyopathy [53,54]. Over time, insulin resistance can cause metabolic disturbances, including increased fatty acid oxidation, lipid accumulation, and inflammation, all of which contribute to cardiac dysfunction [55]. In the context of insulin resistance, the PI3K–Akt pathway is often impaired, leading to disrupted glucose metabolism, increased lipid accumulation in cardiac tissue, and altered myocardial function [56,57]. Thus, the dysfunction of the PI3K–Akt pathway contributes significantly to the pathogenesis of insulin resistance and the subsequent development of heart failure. By activating the PI3K–Akt pathway, baicalin may help restore normal insulin signaling, promote glucose uptake, reduce lipid accumulation, and attenuate inflammation in the heart. These actions could potentially improve heart function and mitigate the progression of heart failure. Therefore, baicalin’s ability to regulate the PI3K–Akt pathway offers a promising therapeutic strategy for diabetic cardiomyopathy.

We expected baicalin to significantly reduce cardiac fibrosis under isoprenaline stimulation. β-Adrenergic insult can trigger severe cardiac fibrosis [58,59], which is consistent with clinical observations that overwhelm sympathetic activation, such as Takotsubo syndrome, can cause long-term cardiac fibrosis [60]. Fibroblasts are the major contributors to cardiac fibrosis, and baicalin suppressed the expression of fibroblast markers both in vivo and in vitro. Furthermore, a PI3K agonist reversed the antifibrotic effect of baicalin, indicating that isoprenaline-induced fibrosis is also related to PI3K activation. Like our findings, Xiao et al. reported that baicalin could alleviate pressure overload-induced cardiac fibrosis [61], and Wu et al. reported that a granule whose major component is baicalin could also attenuate fibrosis in spontaneously hypertensive rats [33]. Hence, our data, along with those of previous reports, indicate that baicalin also has antifibrotic effects.

LY294002 is an inhibitor of PI3Kα, PI3Kδ and PI3Kβ. The inhibition of both the p85a and p110 subunits of PI3K by baicalin combined with LY294002 had a stronger cardioprotective effect than baicalin alone. Specifically, this was reflected in a greater ejection fraction, smaller cardiomyocyte cross-sectional area, fewer markers of myocardial injury, and fewer markers of cardiac fibrosis. These results suggest the potential for baicalin combined with LY294002 to serve as a more effective treatment strategy for HF. However, considering the numerous adverse effects associated with PI3K inhibition by LY294002 [62,63], further experiments are necessary to explore the safe therapeutic dosage of baicalin combined with LY294002.

There were several limitations of our work. First, we used only specific antagonists and agonists of PI3K but not gene silencing or overexpression in the NRCM or NRCF model, which could not exclude the possible off-target effects of these low-molecular-weight drugs. Since PI3K knockout leads to perinatal lethality in mice, we were unable to further research the mechanism of baicalin at the animal level through PI3K knockout. In future research, long-term clinical trials evaluating an effective and safe dose of baicalin combined with LY294002 would be meaningful. In addition to these limitations, our work indeed showed that baicalin was effective in alleviating isoprenaline-induced heart dysfunction and pathological features, indicating that baicalin combined with LY294002 could be a potential supplement to current medical treatments for heart failure with a reduced ejection fraction.

In conclusion, our work revealed that baicalin can ameliorate heart dysfunction and pathological features, including cardiomyocyte hypertrophy and cardiac fibrosis. Baicalin inhibits p85a, which may interact with its SH3 domain to exert its protective effect.

## Data Availability

The data presented in this study are available in PubChem at https://pubchem.ncbi.nlm.nih.gov/ (accessed on 30 May 2023), reference number 64982, and in RCSB PDB at https://www.rcsb.org/ (accessed on 30 May 2023), reference number 8DCP.
